# An approach to rapidly assess sepsis through multi-biomarker host response using machine learning algorithm

**DOI:** 10.1038/s41598-021-96081-5

**Published:** 2021-08-19

**Authors:** Abha Umesh Sardesai, Ambalika Sanjeev Tanak, Subramaniam Krishnan, Deborah A. Striegel, Kevin L. Schully, Danielle V. Clark, Sriram Muthukumar, Shalini Prasad

**Affiliations:** 1 Department of Computer Engineering, University of Texas at Dallas, 800 W. Campbell Rd., Richardson, TX USA; 2 Department of Bioengineering, University of Texas at Dallas, 800 W. Campbell Rd. BSB 11, Richardson, TX USA; 3 EnLiSense LLC, 1813 Audubon Pondway, 1813 Audubon Pond Way, Allen, TX 75013 USA; 4 Austere Environments Consortium for Enhanced Sepsis Outcomes (ACESO), Henry M. Jackson Foundation for the Advancement of Military Medicine, Bethesda, MD 20817 USA; 5 Biological Defense Research Directorate, Naval Medical Research Center-Frederick, Ft. Detrick, MD 21702 USA

**Keywords:** Prognostic markers, Diagnosis, Computational models, Machine learning

## Abstract

Sepsis is a life-threatening condition and understanding the disease pathophysiology through the use of host immune response biomarkers is critical for patient stratification. Lack of accurate sepsis endotyping impedes clinicians from making timely decisions alongside insufficiencies in appropriate sepsis management. This work aims to demonstrate the potential feasibility of a data-driven validation model for supporting clinical decisions to predict sepsis host-immune response. Herein, we used a machine learning approach to determine the predictive potential of identifying sepsis host immune response for patient stratification by combining multiple biomarker measurements from a single plasma sample. Results were obtained using the following cytokines and chemokines IL-6, IL-8, IL-10, IP-10 and TRAIL where the test dataset was 70%. Supervised machine learning algorithm naïve Bayes and decision tree algorithm showed good accuracy of 96.64% and 94.64%. These promising findings indicate the proposed AI approach could be a valuable testing resource for promoting clinical decision making.

## Introduction

Our understanding of the immunology and molecular pathobiology of sepsis has evolved in recent years. The previous understanding of sepsis’s hemodynamic manifestations was primarily related to the hyperimmune host response to a pathogen^1^. A broad range of molecular sepsis research shows an intricate and complex relationship between the host and the infectious agent resulting in heterogenous sepsis manifestations^2^. Cytokines are key indicators of host immune response^3^. Hence, understating the holistic cytokine response in a near-patient setting will be paradigm-shifting in sepsis treatment, enabling better patient stratification towards evidence-based clinical management of the disease^4^. Sepsis etiology monitoring can be achieved by combining point-of-care diagnostic technologies with machine learning (ML) to enable a rapid prognostic understanding of the disease. This information can be used for individualized decision making leading to personalized treatments.

Evaluating biomarkers specific to the host reaction will help strengthen sepsis protocols by their phase classification while regularly facilitating appropriate treatment and patient care. Key pathological responses have been associated with variation in potential biomarkers such as organ dysfunction (IL-6, and PCT), immunosuppressive phase (HLA-DR, monocyte), and hyperinflammatory state (IL-6 and CRP)^5^. Likewise, the relevance of the expression of cell surface markers such as CD64 and CD11b has also been linked to the progression of sepsis, while pro-inflammatory cytokines like TNFα, IL-1, and IL-6 are released in the hyper-immune state of the host^6^. Pro-inflammatory cytokines cause activation and proliferation of leukocytes, activation of the complement system, upregulation of endothelial adhesion molecules and chemokine expression, tissue factor production, and induction of hepatic acute phase reactants^7^. During sepsis, the immune reaction is exacerbated, resulting in collateral damage and healthy cells and tissues' degradation. Hence, the key pro-inflammatory cytokines implicated in the host response of the disease are IL-6, IL-8, IP-10 and TRAIL and the anti-inflammatory cytokine IL-10. To date, all the point of care technology development work involves the characterization of a single or dual host response biomarker with an emphasis on biochemistry^1,8,9^. To have an actionable impact on patient stratification, the implications of the ensemble of host response biomarkers need to be determined, which can be achieved by applying machine learning to result from the multi-marker point of care biosensor devices. This work has looked at five host response biomarker combinations towards providing sepsis prediction relying on evidence-based disease management.

Machine learning has been emerging as a promising tool to identify septic patients and prescribe appropriate antibiotics based on the patient's immune status with good diagnostic accuracy^10^. Several published epidemiological studies have already revealed the risk factors of sepsis^11^. Indeed, a significant number of studies have also reported machine learning to detect and predict the onset of sepsis using these potential variables such as IL-6^12^, IL-8^13^, IL-10^14^, IP-10 and TRAIL^15^. However, to date there has been limited work in utilizing machine learning for stratifying patients through the expression profiles of protein biomarkers associated with the host immune response to the disease. Machine learning offers supervised and unsupervised algorithms. Machine learning takes every responsible input parameter and its degree of effect on the output variable. In this work we are utilizing ML in the context of a classification problem as at the output our goal is to classify the patient based on the host biomarker response at a specific time point such as between 0 and 24 h. Ultimately such classification will help build the patient profile and better resource management at the health care system.

**Figure 1:**
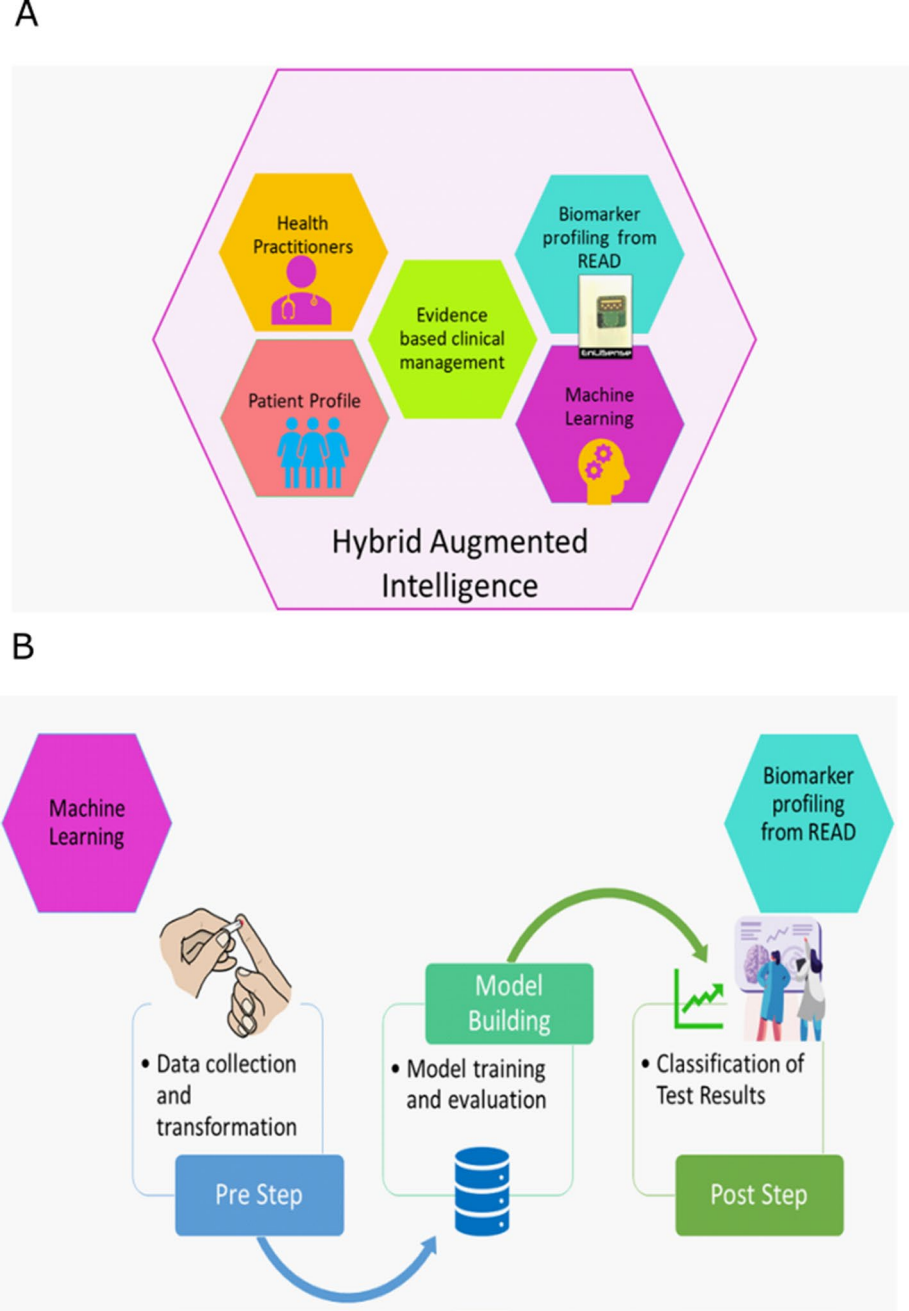
(**A**) Hybrid augmented intelligence approach, (**B**) Machine learning model building process flow.

The system presented in this paper is a combination of the point care system coupled with an intelligent machine learning model. The POC system uses the Rapid Electro Analytical Device (READ) sensor platform enabling swift and reliable sensing of biomarker responsible for studying the host immune response to sepsis. The current commercially available point of care testing methods used for sepsis is focused on identifying pathogen and provides a delayed response. In contrast, the READ platform measures patient’s immune response by quantifying cytokine biomarker levels. The READ platform uses only 40uL of plasma sample and the results are obtained within 5 min. The rapid quantification results will be helpful in better understanding the patient’s condition and providing the specific treatment. The biomarker concentrations are provided as inputs to a machine learning model and the output obtained from the model enables patient stratification. The machine learning model is built with the classification algorithm to minimize errors and maximize the predictions' accuracy. The focus of the manuscript here is building machine learning models capable of patient stratification. This classification will help build an augmented intelligence system suitable for longitudinal patient tracking towards evidence-based clinical management^16^. Figure 1 represents the system hybrid augmented intelligence approach and its components. The idea behind the system is to facilitate a way for health practitioners to take data-driven decisions based on the patient profiles coupled with the biomarker levels obtained from READ integrated with machine learning model outcomes. Together biomarker profiling with machine learning provides the premise of evidence-based clinical management. Figure 1B illustrates the machine learning process which is the focus of the manuscript. The machine learning pipeline is divided into three stages of implementation. The pre-model building step, model building step, and post phase.

## Results

### Biomarker quantification using READ platform

**Figure 2:**
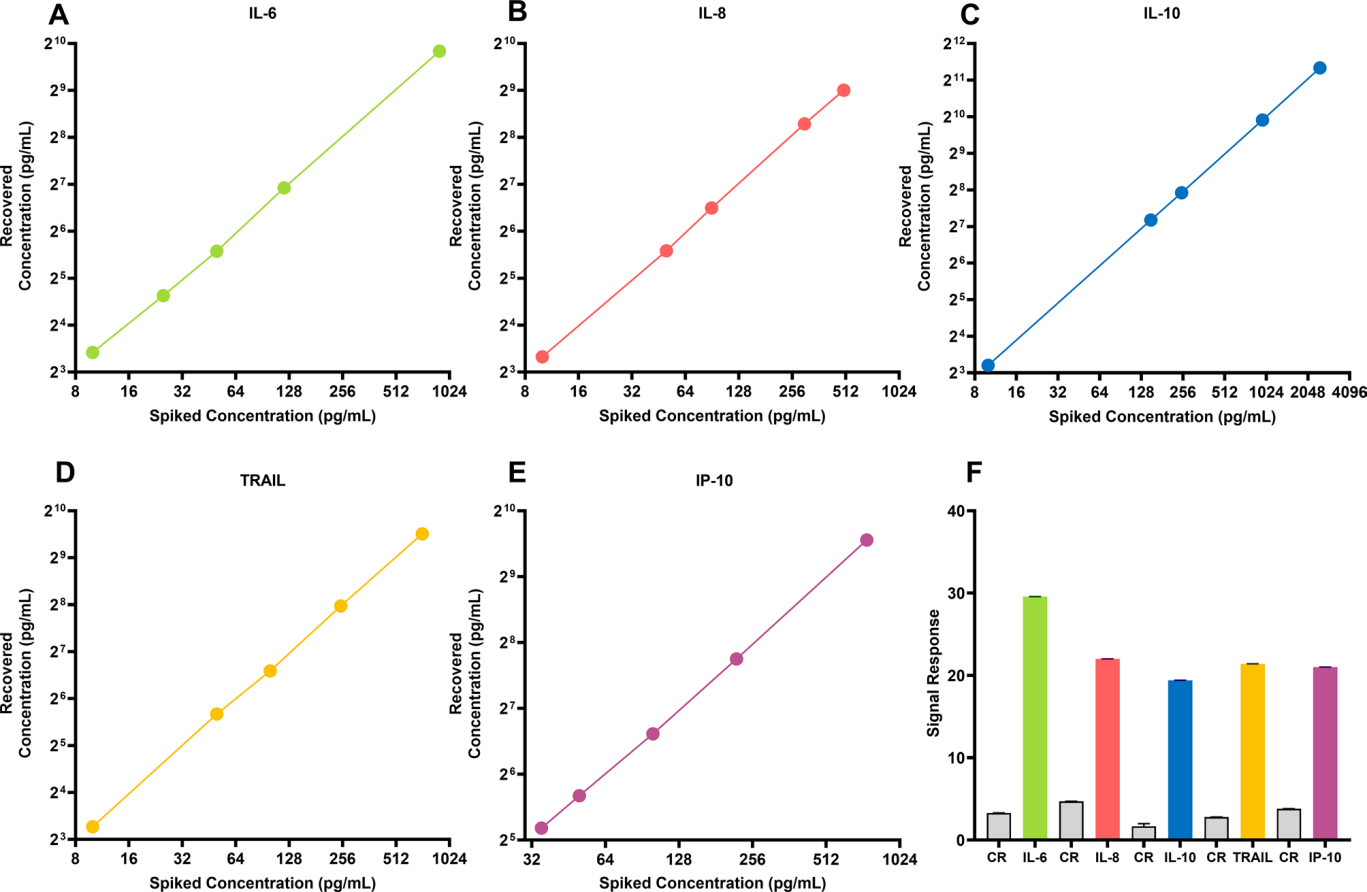
(**A**–**E**) Spike and recovery analysis using READ platforms for all the study biomarkers with a wide dynamic range. (**F**) Cross-reactive (CR) study demonstrates the specificity of the READ sensor platform for IL-6, IL-8, IL-10, TRAIL and IP-10. Data represented in the graphs with n = 3 replicates.

Multiplexed cytokine detection using POC technology is the key to understand sepsis prognosis enabling patient stratification accurately and rapidly. The technology’s biologically applicable performance metrics, combined with the ability to perform multiplexed detection, allows the sensor for clinical diagnosis based on inflammatory cytokines panel^17^. READ sensor platform was evaluated based on spike and recovery study. The sensor surface was immobilized with specific capture antibodies. Varying target analyte doses were spiked in pooled human plasma and signal response from each was quantified using electrochemical impedance spectroscopy. Figure 2A–E demonstrates the READ sensor platform's capability to detect spiked concentration accurately with a coefficient of variation < 12%. The average recovery for IL-6, IL-8, IL-10, IP-10 and TRAIL was between ~ 80 and 120%, according to the clinically acceptable limit^18^. One-way ANOVA was used to determine if the group means for each of the spiked concentrations were statistically significant from one another. P-value < 0.0001 with a confidence interval of 95% between the spiked doses indicates the reliability in detecting each dose concentration in pooled human plasma for IL-6, IL-8, IL-10, TRAIL and IP-10. High specificity is crucial for a biosensor, especially while testing patient samples in a complex buffer medium^19^. Thus, we assessed the specificity of the biosensor to common cross-reactive biomarkers for every analyte. Each capture probe was tested with high doses of cross-reacting molecules and measured in triplicate. As seen in Fig. 2F, the results indicate the biosensors' ability to detect target analytes with high specificity for IL-6, IL-8, IL-10, TRAIL and IP-10 in pooled human plasma.

### Study protein exploratory data analysis

**Figure 3:**
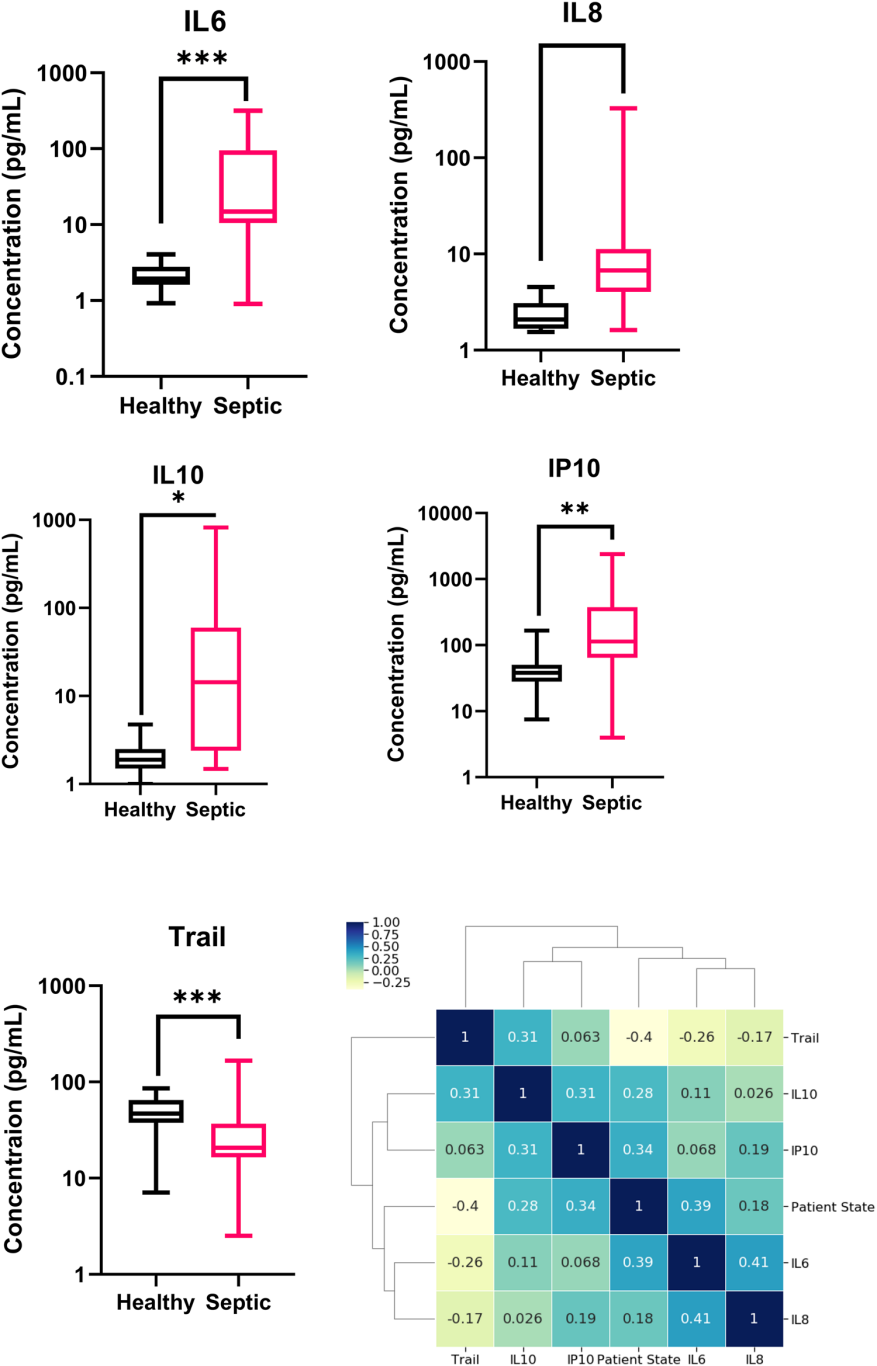
(**A**–**E**) Comparing the healthy control vs septic patient samples for IL-6, IL-8, IL-10, IP-10 and TRAIL. Significance *p < 0.1, **p < 0.01, ***P < 0.001. (**F**) Correlation matrix with heatmap and cluster map showing relation between the study biomarkers and patient state.

Figure 3 describes the characteristics of the dataset such as trends, patterns and relationships between variables. This manuscript focuses on the proof-of-feasibility demonstration of patient classification based on the combination of the measured cytokine biomarkers. Figure 3A–E have the concentration of study protein level mapped on the y-axis and the x-axis represents the patient state. All the biomarkers show the distinct boundary of classification. TRAIL biomarker showed downregulation compared to the healthy cohort as shown in previous study^20^. Significance was obtained when a t-test was performed between the healthy and septic group, further distinguishing the two data sets for the study biomarkers. The last figure in the set Fig. 3F combines the correlation matrix presented with the cluster map. The correlation matrix represents the associated relationship between the study proteins, qSOFA scores and patient state. Although correlation does not confirm the causation, it enables framing the naïve assumption. Data interpretation of the correlation matrix is visualized using the color gradient on the heatmap. Herein, blue represents positive correlation, yellow indicates the negative correlation and color shade indicates the strength of correlation. The highest degree of correlation seen in the figure was 0.39 between the patient state and IL-6 biomarker except for self-correlation. Considering the correlation with patient state IL-6 is followed by IP-10, IL-10 and IL-8. However, TRAIL biomarker showed a negative correlation with the patient state due to the downregulation seen in the septic patient cohort (Fig. 3E). Therefore, the correlation matrix heatmap confirms the single study protein is not enough evidence to determine the patient's septic condition. This inference is the primary intention behind going for a multiple study protein approach rather than single.

### Evolution of machine learning model

**Figure 4:**
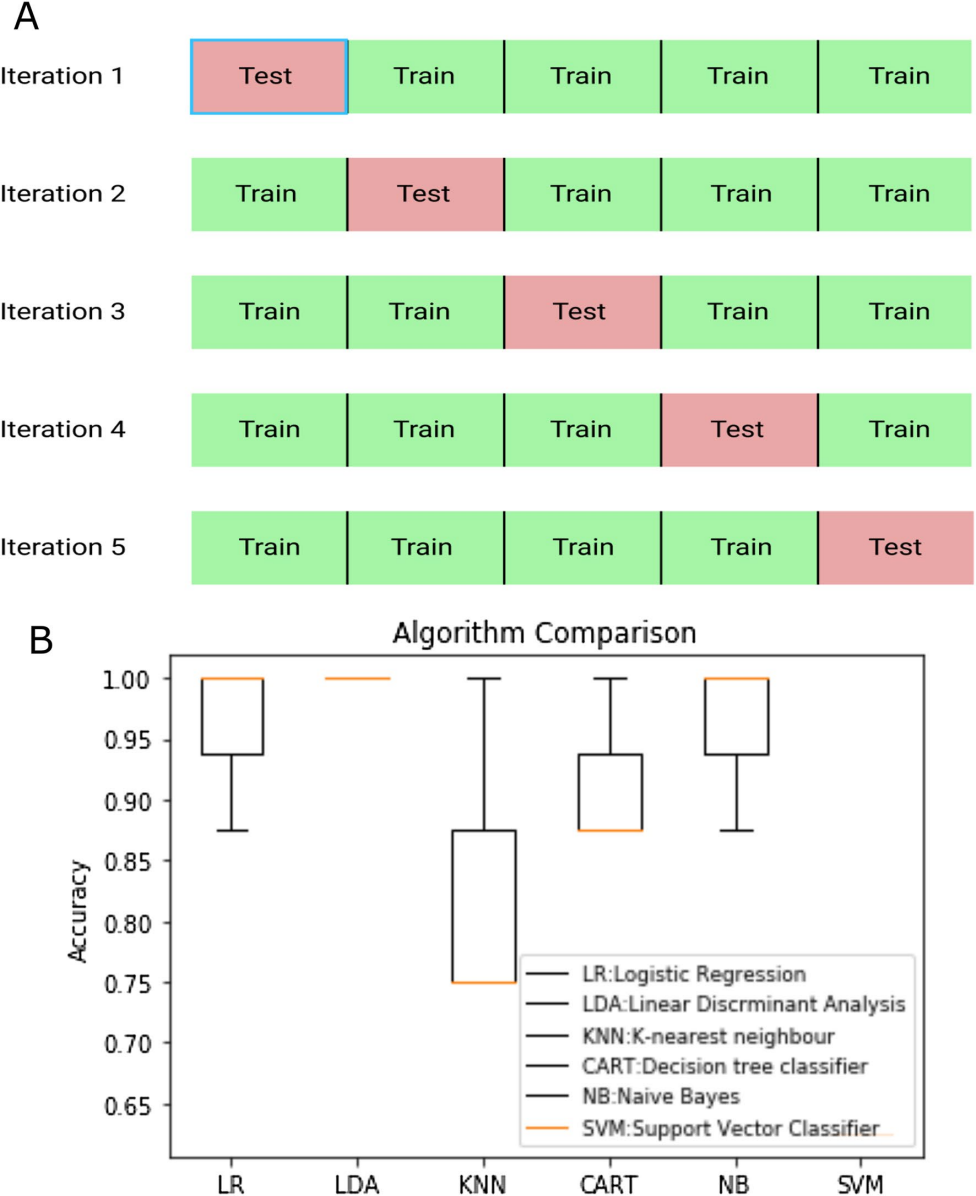
(**A**) k-fold validation method with illustration of k = 5, (**B**) is the comparison from k-fold validation of linear regression(LR), Linear Discriminant Analysis(LDA), k-Nearest Neighbor (KNN), Classification and Regression Tree(CART), Naïve Bayes(NB), Support Vector Machine(SVM).

Figure 4A represents the k-fold method where the execution was done in the iteration manner and the results are represented as box plots for the visual comparison in Fig. 4B. K value was chosen as 5 for this study. The dataset size was comparatively small, hence k = 5 provided a robust outcome. K-fold validation helps to build confidence in the model performance. Figure 4B captured the essence of all 5 iterations with the methodology they were executed. The summary of iteration accuracy is plotted as the box plot for comparison. Every time the test and validation dataset were varied, thereby altering the performance. Accuracy was used as a metric to validate the machine learning models. Mean accuracy was calculated for each algorithm post iterations. The algorithms with their respective accuracies in shown in supplementary (Table S1). 95% accuracy was observed with the decision tree (CART) algorithm. This was followed by Naïve Bayes and logistic regression (92%), K-nearest neighbors algorithm (k = 3) showed 84% accuracy whereas support vector classifier demonstrated the least accuracy of 63%.

### Naïve Bayes and Decision tree

**Figure 5:**
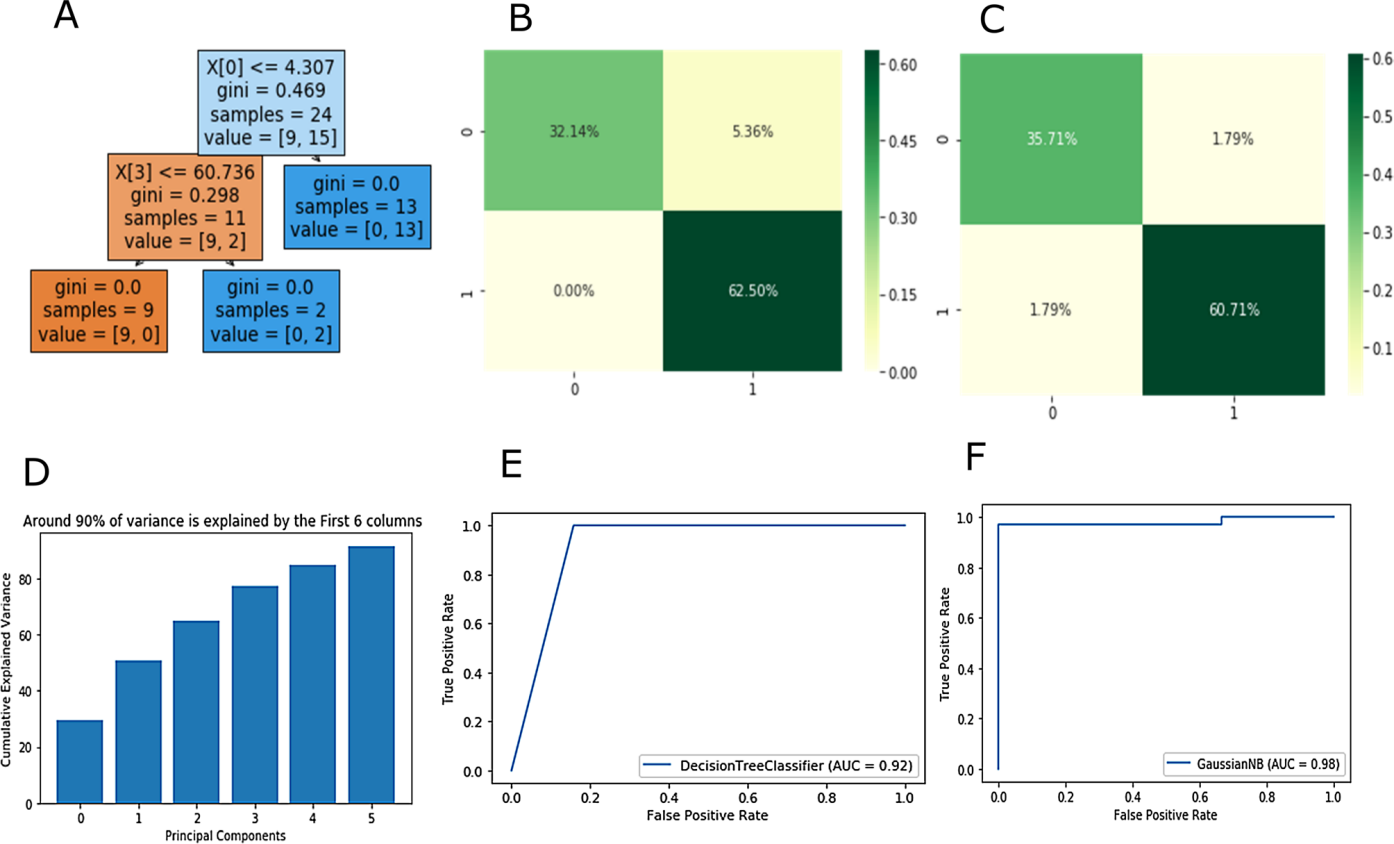
(**A**) Decision tree evolution using CART algorithm, (**B**) Confusion matrix for Decision Tree (CART), (**C**) Confusion matrix for Naïve Bayes, (**D**) Principal component analysis showing 90% variance by first 6 parameters that are biomarkers, (**E**) AUCROC curve for Decision Tree, (**F**) AUCROC curve for Naïve Bayes.

Based on k-fold validation results, Naïve Bayes and Decision Tree (CART) algorithm was down selected as they demonstrated high accuracy. Figure 5 represents the selected algorithms performance. Figure 5A shows the evolution of the decision tree. At every iteration of splitting, the best input feature was selected from the remaining input parameters, initially, all the study protein levels were used as inputs. In the first iteration, the input feature X [0] was IL-6 selected at first split as this has the highest degree of correlation with the output patient state. The threshold value was based on the training values and representative of the training set. The training data was stratified to avoid class imbalance effect. The accuracy of the training dataset was found to be 91.16% which shows room for generalization.

Figure 5B shows the confusion matrix for the decision tree algorithm. For the binary classification task, the confusion matrix was divided into the 4 parts; True Positive (TP), True Negative (TN), False Positive (FP), and False Negative (FN). False-positive and false-negative values give rise to type I and type II errors, respectively, which were to be minimized in implementation. Both the errors have their own disadvantages and we have done our efforts to keep it minimal. The number of TP and TN are contributed towards the accuracy. The FP in other terms the type I error was 5.36% for the given implementation. In medical diagnosis, the type I error is less significant as compared to type II error. The type II error or FN was found to be 0% in the implementation. Although the algorithm's performance is subject to the available data, the overall accuracy achieved with the decision tree algorithm was 94.64%.

Similarly, a confusion matrix for the naïve Bayes algorithm is represented in Fig. 5C. The naïve Bayes confusion matrix shows the presence of both type I and II errors. Each contributes to 1.79% of the total. The accuracy which combines the TP and TN rates was calculated to be 96.64%. In comparison with the decision tree's performance, naïve Bayes shows slightly higher accuracy with a reduced total error of 3.6%. Despite the advantages, it shows the presence of type II error (1.79%), which is relatively low. Overall, both algorithms show similar performance and serve the purpose of classification. Choice can be made depending on the objective of the study.

Figure 5D explains the variance of the dataset with the help of principal component analysis. To retain 90% of the original variance 6 parameters are needed. The most dominant components are considered first and the contribution of each decreases gradually. Figure 5E,F represents AUC (Area Under the Curve) ROC (Receiver Operating Characteristics) curves. This signifies the sensitivity and specificity of the model. A high AUC value represents the ability of the model to predict the classes as true they are. The AUC curve for the decision tree was 0.92 and for naïve Bayes at 0.98. High accuracy indicates a higher ability of the models to differentiate between septic and healthy patient state. The AUC = 1 represents the perfect classifier. Overall, the observations suggest a high accuracy of machine learning algorithms using five host response biomarkers may yield better clinical outcomes for sepsis detection.

## Discussion

The exploratory data analysis shows a promising distinguishable boundary between the septic and healthy cohort for all the study biomarkers. Gender bias is not seen in any study protein levels hence we can say the gender parameter will not affect the model's outcome to much extent. The highest degree of correlation between study proteins and patient is seen to be 0.39 which alone is not enough to conclude the patient's state. The study's objective was to build an accurate model to accurately predict a given biomarker level into septic or healthy cohort. The choice of input parameter was based on principal component analysis (PCA). PCA showed that the dataset variance was highly dependent on the study protein levels and the qSOFA value. The final model takes account of demographic input parameters age and gender. The summary of demographic information has been added in the supplementary Table S3.

As the dataset available is limited, we used k-fold validation to gauge the model’s true performance over different training sets. All the k times the training dataset was different from each other and tested on previously unexposed validation set to the model. Having a different set of training and testing set reduces the bias of the model. This way every observation in the dataset can be treated as a part of the training and testing set, compensating for the limited datapoint and reducing the variability of the model. The accuracy obtained for the iteration has been logged and average accuracy as considered for the final algorithm selection. The k-fold method was used to get more confidence at the algorithm selection stage. The results obtained from the average value of all iteration is used for finalizing the algorithm for the study. The accuracy of the algorithm was the main criteria for selection. The selected algorithm is further fine-tuned to attain better accuracy and lower the type I and II errors. The classifier decision tree (CART) and naïve Bayes were the top performers in k-fold validation method. Hence, they were further implemented to validate the performance on the test data set. In CART algorithm, at each split, we were able to achieve the pure node and categorize them in the correct bin. The model's robustness can be confirmed based on the confusion matrix for both algorithms as they displayed type I and II errors below 6% (maximum was 5.36%).

Our study has a few limitations. Firstly, the scope of the research is limited to differentiating healthy and septic patients. The machine learning model can differentiate between the septic and healthy patient accurately, but it cannot distinguish between the severity of the patient’s state in terms of sepsis. The model presented here is also susceptible to limited dataset size. Currently, the data size is small, and few adjustments will be needed to get the large-scale working model on real-time data. Another limitation with the small dataset is that model is prone to overfitting. The overfitting can be avoided if there is room for generalization without hampering the accuracy. Our model minimizes overfitting and it can be confirmed from ROC curve. Our ongoing study involves patient information from multiple time points during patient stay during sepsis to build a better sepsis prediction model. The time-point study of sepsis will help provide more significant insights about sepsis and the urgency to seek treatment. The future goal is not just to limit the study for the prediction of sepsis but also to predict its severity.

Small sample size limits machine learning algorithms and may suffer from issues of interpretability based on the available variables. However, compared with similar algorithms such as SVM, KNN and LDA, naive Bayes and the decision tree algorithm provide better outcomes. In conclusion, the results demonstrate the application of machine learning algorithms that can be used to predict sepsis in patients with high accuracy and become a screening tool to support clinical decisions. Additional research is required to evaluate improvements in predictive outcomes by increasing the sample data set and evaluating other relevant patient input features. The developed approach serves as an illustration that can be generalized and implemented to include other variables such as integrating electronic health records to enable improved clinical forecasts for sepsis patients.

## Methods

### Sensor technology

The READ platform encompasses a portable, single-use sensor with an array of sensing electrodes independently configured to measure multiple biomarkers concurrently from the target sample in real-time. Capture probes specific to target analytes are functionalized on the array of electrodes to allow multiplexed detection. Electrochemical impedance spectroscopy captures the subtle changes between a specific capture antibody and the target analyte with a measurable impedance response at the electrode surface. Cytokine biomarker panel was used with the READ platform for the detection of Interleukin 6 (IL-6), interleukine-8 (IL-8), interleukin -10 (IL-10), and interferon-inducible protein (IP-10) and tumor necrosis factor-related apoptosis-inducing ligand (TRAIL) in blood plasma.

READ device has 2 components 1. Single-use sensor functionalized to detect multiple cytokine biomarkers simultaneously 2. A palm size reader for sensor mounting. The small size and single-use of the sensor make READ a point-of-care device. Electrochemical Impedance Spectroscopy (EIS) has been used to translate impedance into human-readable measurement value of measure cytokine biomarker. The device operates with battery and can be able to have a wired as well as a wireless interface for signal transfer. The device uses a simple easy to follow user interface for the operation of the device. This cuts the training cost of specialized personal. It also reduces human error and variability that might affect results during the handling of the samples^21^.

### Patient sample acquisition

Plasma samples were collected under written informed consent as part of an ongoing observational trial of sepsis in resource-limited settings conducted by ACESO^22,23^. Briefly, adult patients presenting to a participating hospital's emergency department with suspected infection (as judged by the attending physician) and met at least two of three SIRS clinical criteria (SEPSIS-2 criteria) were eligible for enrollment. For this work, patient plasma samples collected 24 h after enrollment were used. Plasma samples from n = 50 patients were provided stripped of all identifiers by ACESO to Biomedical Microdevices and Nanotechnology Laboratory, UT Dallas, according to the Material Transfer Agreement (MTA), approved by the Institutional Review Board (IRB# 19MRO151) at the University of Texas at Dallas. De-identified patient information such as gender, age, location, along with the QSOFA score assigned at the time of admission, was used for data analysis. Plasma samples from healthy individuals were used as control samples for n = 30. All the methods were carried out in accordance with clinical guidelines and regulations.

Multiplexed cytokine (Il-6, IL-8, IL-10, TRAIL and IP-10) concentrations measured by READ platform along with the patient age are positive numbers. Whereas gender information and location are classified as categorical variables. For cytokine measurement READ platform used 40uL of plasma sample for all the experiments. Graphpad software (GraphPad Software Inc., La Jolla, CA) was used to analyze the data and calculate the cytokine concentration. Additional patient information with underlying conditions can be found in Supplementary Table S3. Data analysis was performed using a total of 80 samples, where 50 samples were classified as a septic cohort and the remaining 30 as a healthy cohort. Patient data collected by READ platform was segregated into training and test groups. To build a robust model, 70% of data was used as training data set, while 30% of data was as test data set.

### Descriptive data analysis

This type of analysis lays the foundations of the analysis. The box plot here is used to portray the patient classification in the dataset. The advanced method to get insight from the dataset is the correlation matrix. The correlation matrix is the matrix where the intersection of the row and column gives the correlation coefficient. In order to calculate the degree of correlation, we have used Pearson’s coefficient of correlation given by the formula

1 $${r}_{xy}=\frac{n\sum {x}_{i}{y}_{i}-\sum {x}_{i}\sum {y}_{i}}{\sqrt{n\sum {x}_{i}^{2}-{(\sum {x}_{i})}^{2}}\sqrt{n\sum {y}_{i}^{2}-{(\sum {y}_{i})}^{2}}}$$

rxy = Pearson r correlation coefficient between x and y, n = number of observations, xi = value of x (for ith observation), yi = value of y (for ith observation).

### Machine learning

The study aims to assess the host response using machine learning for the classification of the patient. Various algorithms are available for supervised classification algorithms, such as logistic regression, naïve Bayes, k-nearest neighbor, decision tree and support vector machine^24^. Given the limited dataset we chose the simple to complex algorithm for the implementation. Logistic regression, naïve Bayes is a better option for the linearly separable data. A decision tree works well when input parameters have a distinguishable boundary between the output classes. We have seen decision boundary within cytokine concentration as well as in the PCA performed we choose these algorithms. We also wanted to make sure we consider the boundary point and their correct classification we considered an option of k-nearest neighbor and support vector machine algorithm. Based on an algorithm's performance during k-fold testing, the best two methods were selected for further implementation and fine-tuning.

K-fold cross-validation process adds the additional validation phase during the training phase itself. The training process is broken down into k iterations. The training dataset is divided into the k sections. At every iteration (k-1) section are used for the training and the remaining one is used as the internal validation set. The performance of the algorithm is noted for the iteration. The same is repeated for the k items every time the validation set is different. The performance of the algorithm is aggregated for comparison.

The developed models are further analyzed for the robustness and generalizability criteria. It is achieved by the sensitivity–specificity graph and the confusion matrix criteria. The hypothesis was supported using the READ cytokine biomarker results.

## Supplementary Information

Supplementary Information.
