# Supplementary material for: An approach to rapidly assess sepsis through multi-biomarker host response using machine learning algorithm
Source: Sci Rep. 2021 Aug 19;11:16905. doi: 10.1038/s41598-021-96081-5 (PMC8377018; doi:10.1038/s41598-021-96081-5)
Supplement: Supplementary file 1 — Supplementary Information. [file 41598_2021_96081_MOESM1_ESM.docx]

An approach to rapidly assess sepsis through multi-biomarker host response using machine learning algorithm

Abha Umesh Sardesai^1^, Ambalika Sanjeev Tanak^1^, Subramaniam Krishnan^3^, Deborah A Striegel^3^, Kevin L Schully^4^, Danielle V Clark^3^, Sriram Muthukumar^1,2^, Shalini Prasad^1,^*

^1^Department of Bioengineering, University of Texas at Dallas, Richardson, TX 75080, USA

^2^EnLiSense LLC, 1813 Audubon Pondway, Allen, TX 75013, USA

^3^Austere environments Consortium for Enhanced Sepsis Outcomes (ACESO), Henry M. Jackson Foundation for the Advancement of Military Medicine, Bethesda, MD 20817, USA

^4^Biological Defense Research Directorate, Naval Medical Research Center-Frederick, Ft. Detrick, MD 21702, USA

***Corresponding Author(s): Shalini Prasad**

Address: 800 W. Campbell Rd. BSB 11,

Richardson, TX, USA 75080

Email: [shalini.prasad@utdallas.edu](mailto:shalini.prasad@utdallas.edu)

Phone: 972-883-4247

Sriram Muthukumar

Address: 1813 Audubon Pond Way,

Allen, TX, USA 75013

Email: [sriramm@enlisense.com](mailto:sriramm@enlisense.com)

**Supplementary Information**

## Algorithm comparison for k-fold comparison

| Algorithm | Mean Accuracy |
| --- | --- |
| Decision Tree Classifier | 95% |
| Naïve Bayes | 92% |
| Linear Regression | 92% |
| k-Nearest Neighbor | 84% |
| Support Vector Machine | 63% |

S.Table 1: summary of mean accuracies found through k-fold method for classification algorithm considered for the study

## Descriptive Analysis of biomarkers

Healthy Group


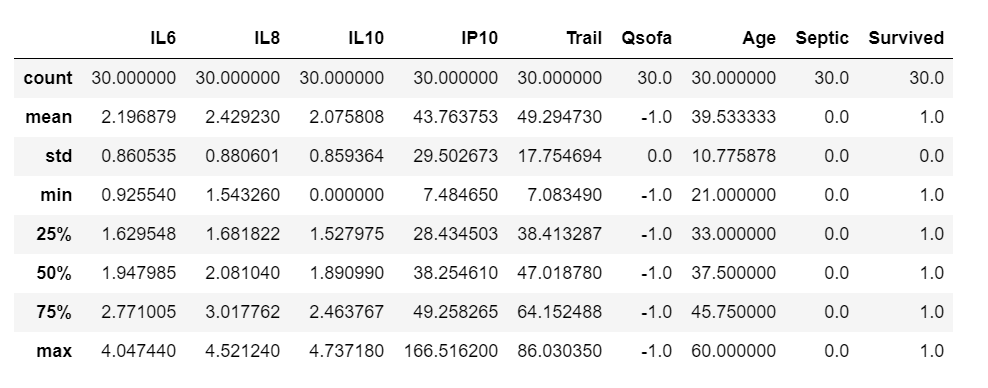


Septic Group


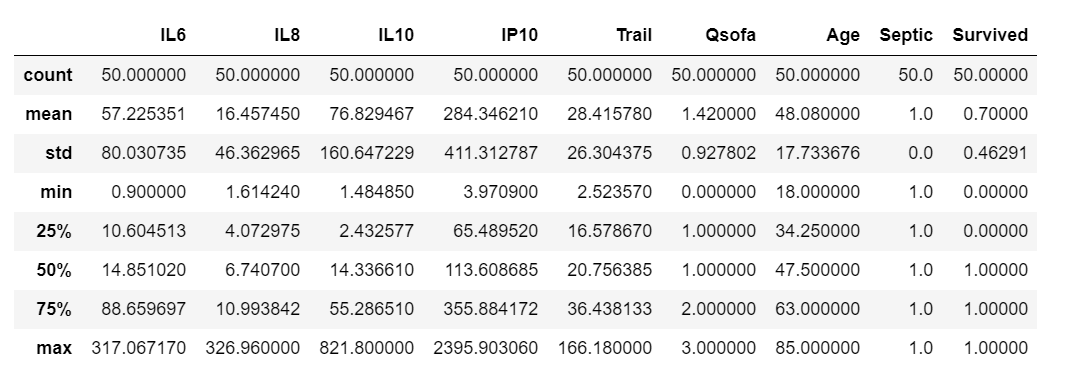


S.Table 2: Descriptive statistics about the biomarkers and qsofa

## Correlation matrix with Cluster and heat mapping with all the parameters


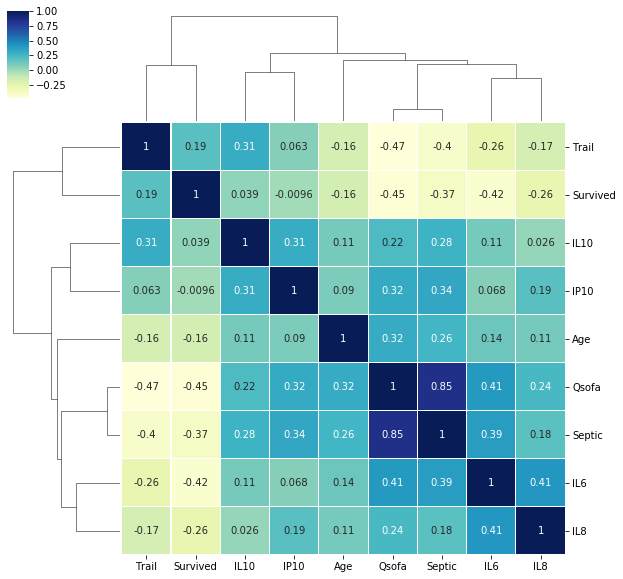


## Patient characteristics data shown as group mean

| **Characteristic** | **Value** |
| --- | --- |
|  | **(IQR)** |
| age (Range) | 48 (32-63) |
| Gender (Male/Female) | 26/21 |
| qSOFA score | 2(0-3) |
| **Site of infection** |  |
| Lower respiratory | 14 |
| Other | 7 |
| Systemic | 6 |
| Dermatology | 6 |
| Gastrointestinal (GI) | 5 |
| Genitourinary (GU) | 3 |
| Central Nervous system (CNS) | 3 |
| Head, eyes, ears, nose & Throat (HEENT) | 2 |
| Intermediate | 1 |
| Unknown | 3 |
| **History** |  |
| Respiratory | 11 |
| Immunosuppression | 7 |
| Endocrine | 17 |
| Other | 16 |
| Psychiatric | 15 |
| Cancer | 6 |
| Cardiovascular | 17 |
| Neurological | 5 |
| Gastrointestinal | 4 |
| Renal | 2 |
| Gu or reproductive | 2 |
| Rheumatologic | 3 |
| Surgery | 2 |
| Lactate avg | 2.53 (1.20) |
| wbc | 14.39 (7.85) |
| temp | 38.14(1.11) |

S.Table 3:Summary about patient profiles presented as group meanss
